# Leveraging Landmark Analysis for Tailored Surveillance in Stage I Non-Small-Cell Lung Cancer †

**DOI:** 10.3390/cancers18030367

**Published:** 2026-01-24

**Authors:** Giovanni Leuzzi, Federica Sabia, Matteo Calderoni, Clarissa Uslenghi, Ugo Pastorino, Alfonso Marchianò, Michele Ferrari, Alessandro Pardolesi, Daniele Lorenzini, Giuseppe Lo Russo, Claudia Proto, Arsela Prelaj, Piergiorgio Solli

**Affiliations:** 1 Division of Thoracic Surgery, Fondazione IRCCS Istituto Nazionale dei Tumori, 20133 Milan, Italy; federica.sabia@istitutotumori.mi.it (F.S.); matteo.calderoni@istitutotumori.mi.it (M.C.); clarissa.uslenghi@istitutotumori.mi.it (C.U.); michele.ferrari@istitutotumori.mi.it (M.F.); alessandro.pardolesi@istitutotumori.mi.it (A.P.); piergiorgio.solli@istitutotumori.mi.it (P.S.); 2 Lung Cancer Prevention Research, Fondazione IRCCS Istituto Nazionale dei Tumori, 20133 Milan, Italy; ugo.pastorino@me.com; 3 Department of Radiology, Fondazione IRCCS Istituto Nazionale dei Tumori, 20133 Milan, Italy; alfonso.marchiano@istitutotumori.mi.it; 4 Department of Diagnostic Innovation, Fondazione IRCCS Istituto Nazionale Dei Tumori, 20133 Milan, Italy; daniele.lorenzini@istitutotumori.mi.it; 5 Department of Oncology and Hemato-Oncology, University of Milan, 20122 Milan, Italy; 6 Department of Medical Oncology, Fondazione IRCCS Istituto Nazionale dei Tumori, 20133 Milan, Italy; giuseppe.lorusso@istitutotumori.mi.it (G.L.R.); claudia.proto@istitutotumori.mi.it (C.P.); arsela.prelaj@istitutotumori.mi.it (A.P.)

**Keywords:** NSCLC, early stage, surveillance, CT scan, new primary lung cancer

## Abstract

**Background**: Current guidelines for NSCLC follow-up lack specific recommendations on surveillance duration. This study aims to analyze survival and surveillance data in resected stage I NSCLC. **Methods**: We retrospectively reviewed 759 pathological stage I NSCLC (9thTNM ed.) patients with no history of lung cancer (LC) undergoing surgery from January 2003 to December 2018. Overall survival (OS), incidence of relapse (IR), and incidence of new primary LC (NP) were analyzed. Long-term effect of follow-up beyond 5 years was assessed by landmark analysis of OS, IR, and NP at 10 years, restricted to individuals alive without relapse or NP at 5 years (5-year event-free survivors, 5y-EFSs). **Results**: The rates of 10-year OS, 10-year IR, NP incidence, and 5y-EFSs were, respectively, 75%, 18%, 1.1%/year, and 59.1% (449 patients). Carcinoid IA/IB (0–10%) and adenocarcinoma IA/IB without lung nodules (LNs) (8–12%) had a similarly lower risk of relapse (*p* = 0.5088) compared to adenocarcinoma with LNs (*p* = 0.0191). Similarly, carcinoid (0–0.2%/year) and adenocarcinoma without LNs (0-0.3%/year) had the same lower incidence of NP (*p* = 0.8062) compared to patients with LNs (*p* < 0.0001). The group of 5y-EFSs had a conditional 10-year OS, IR, and NP incidence of 92%, 5%, and 0.8%/year. In 5y-EFSs, 10-year OS was better in carcinoid (100%) and adenocarcinoma (94%, *p* = 0.0009) patients; 10-year IR was lower in stage IA (4%) vs. IB (10%, *p* = 0.0444), and NP was lower in patients with no pre-surgery (0.5 vs. 1.5%/year, *p* = 0.0147) and no post-surgery LNs (0.6 vs. 1.1%/year, *p* = 0.0202). **Conclusions**: Based on our results, we propose a tailored surveillance strategy by de-escalating follow-up for low-risk patients while maintaining intensive monitoring for high-risk individuals.

## Simple Summary

This study investigated the long-term outcomes and surveillance patterns in 759 patients who underwent surgery for pathological stage I Non-Small-Cell Lung Cancer (NSCLC). We sought to provide evidence to refine current follow-up guidelines, which often lack specific recommendations regarding the optimal duration of surveillance after curative resection. Crucially, the study found that histology and the presence of lung nodules (LNs) were key determinants of late events. Patients with carcinoid tumors and those with adenocarcinoma without LNs demonstrated a significantly lower risk of relapse and new primary tumors compared to high-risk groups. Focusing specifically on 5-year event-free survivors (5y-EFSs), these patients exhibited excellent conditional 10-year OS (92%), but the risk of late events persisted for those with stage IB disease or pre-existing/new LNs. Based on these findings, we propose a tailored, risk-stratified surveillance strategy. This model supports de-escalating follow-up intensity for patients identified as low-risk (such as those with carcinoid and adenocarcinoma without LNs) while ensuring that high-risk individuals receive continued, intensive monitoring to promptly detect potentially curable late events.

## 1. Introduction

Lung cancer (LC) still represents the primary cause of cancer-related mortality globally. It is well-established that complete surgical resection is the gold standard of curative treatment in early-stage Non-Small-Cell Lung Cancer (NSCLC), offering the best prognosis. Post-op follow-up is mandatory for detecting two critical events, namely local/distant recurrence and new primary lung cancer (NP). In this setting, identification of both events is essential, as early intervention is associated with improved long-term outcomes.

Despite the critical nature of post-operative surveillance, international guidelines lack consensus on the optimal duration and intensity of follow-up for resected NSCLC. Major clinical societies, including the National Comprehensive Cancer Network (NCCN), the European Society for Medical Oncology (ESMO), the American Society of Clinical Oncology (ASCO), and the American College of Chest Physicians (ACCP), generally recommend biannual/annual surveillance with low-dose chest computed tomography (CT) scans [1,2,3,4]. However, while continued surveillance beyond 5 years is often suggested, this recommendation is primarily driven by the persistent, lifelong risk of developing NP rather than a high risk of true tumor relapse, particularly in lower-risk cohorts like stage I patients [1,3]. The absence of specific cut-off points for cessation leads to wide discrepancies in clinical practice and potential inefficiencies in resource use.

The traditional 5-year disease-free survival (DFS) period has long been adopted as a proxy for “cure” in NSCLC. However, some authors reported that *late recurrence* (occurring more than 5 years after curative surgery) is not so uncommon and is reported in up to 11% of patients [5]. In addition, *ultra-late recurrence*, occurring beyond 10 years, has also been explored, underscoring the indefinite nature of risk for some patients [6]. This sustained risk of both NP and recurrence underscore the necessity for long-term follow-up, as well as to identify prognostic factors among long-term survivors [7].

To effectively design a tailored surveillance strategy, it is crucial to identify which patients are truly cured at a given time point and which carry a significant, persistent risk of late events. Evaluating the factors influencing patient outcomes conditional on surviving disease-free for a given period, such as 5 years, requires specialized methodology [8].

This study aims to analyze long-term clinical outcomes, including overall survival (OS), incidence of relapse (IR), and incidence of NP (INP), in a large single-institution cohort of completely resected pathological stage I NSCLC patients in order to identify specific patient subgroups who benefit (or not) from continued and intensified surveillance beyond the conventional 5-year period, thereby informing a more efficient and personalized follow-up strategy.

## 2. Patients and Methods

### 2.1. Study Design and Patient Selection

This study constitutes a retrospective analysis of prospectively maintained clinical and pathological data from a dedicated institutional database at Fondazione IRCCS Istituto Nazionale dei Tumori (Milan, Italy). The study was conducted in accordance with the Declaration of Helsinki and approved by the Institutional Review Board (IRB) of Fondazione IRCCS Istituto Nazionale dei Tumori (IRB Protocol No. INT 6-25, approved on 7 February 2025). In accordance with the Italian Privacy Code (D.Lgs. 196/2003, Art. 110-bis, par. 4) regarding the institutional research mandate of IRCCS institutions, and given the retrospective nature of the study, the IRB specifically approved the use of existing de-identified data and granted a waiver for individual re-consent. Patient data confidentiality was strictly preserved throughout the study.

We retrospectively reviewed the records of all consecutive patients who underwent curative-intent surgery for pathological stage I NSCLC according to the 9th edition of the AJCC/IASLC TNM classification between January 2003 and December 2018.

Inclusion criteria were as follows: (1) pathological diagnosis of stage I NSCLC (IA or IB); (2) intention-to-treat surgery; (3) complete surgical resection (R0); (4) absence of prior history of lung cancer or other synchronous malignancies at the time of surgery; and (5) systematic nodal dissection. Patients who received neoadjuvant or adjuvant systemic therapy, or those with R+ resection or incomplete follow-up data, were excluded from the analysis. The final cohort consisted of 759 patients; only 1 patient had an unknown vital status and was excluded from the corresponding analysis.

### 2.2. Surgical and Clinicopathological Data

Relevant clinicopathological variables were extracted from the institutional database, including patient demographics (age, sex, smoking history, pack-years, presence of COPD or other prior malignancies), tumor characteristics (histological subtype, pTNM stage), and type of resection.

### 2.3. Follow-Up Policy

At our institution, all patients with stage I NSCLC undergoing surgical resection are managed through a dedicated “Follow-up and Research” outpatient clinic. Patients in this clinic adhere to a systematic surveillance protocol for up to 10 years, which includes a low-dose chest CT scan and an abdominal ultrasound (US) every 6 months for the first 2 years, followed by an annual chest CT scan and abdominal US until 10 years post-surgery. In the setting of stage I NSCLC only, we utilize abdominal US to adhere to a crucial institutional radiation reduction strategy, aiming to minimize the patient’s cumulative radiation dose over the 10-year surveillance period. During follow-up visits, patients are physically examined, and all radiological images are meticulously reviewed by a thoracic surgeon.

Special attention was given to the assessment of lung nodules (LNs) identified during the surveillance period. The presence of new LNs was diligently evaluated (in complex cases with a specialized radiologist), and their subsequent follow-up was conducted in accordance with the NCCN guidelines [3].

Data access and survival verification for this retrospective analysis were performed by verifying survival status and follow-up data for all patients, including those lost to clinical follow-up, through comprehensive institutional medical records and cross-referencing with the Regional and Italian National Death Registry (Punto Fisco). This process ensured the accuracy and completeness of the long-term survival and event data reported.

### 2.4. Statistical Analysis

Outcome events were defined as follows:

- OS: Calculated from the date of surgery to the date of death from any cause or the last follow-up date.
- IR: Proportion of patients that experience a local, regional, or distant relapse of the original LC (pathologically or radiologically confirmed) within a specific time period (5- and 10-year).
- NP: A subsequent malignant LC pathologically confirmed to be distinct from the index cancer, based on different features in accordance with ACCP guidelines [9]: (1) same histology, temporally separated; or (2) 4-yr interval between cancers and no systemic metastases from either cancer; or (3) different histologic type or different molecular genetic characteristics or arising separately from foci of carcinoma in situ.

Continuous variables were presented as medians and Interquartile Ranges (IQRs), and categorical variables as counts and percentages. OS, IR, and INP curves were estimated at 10 years from the date of surgery using the Kaplan–Meier method (for OS) and cumulative incidence function (CIF) for competing risk events (for IR and INP), respectively. Death from another cause different from the event of interest (non-relapse or non-NP mortality, respectively) was treated as a competing event for IR and INP, as it could prevent the occurrence of relapses or new cancers. Curves were stratified by selected variables (e.g., stage, histology, presence of LNs) and differences were tested using the Log-Rank test (for OS) and Gray’s test (for IR and INP).

The long-term effect of follow-up beyond 5 years was evaluated using landmark analysis at the 5-year time point. This analysis was restricted to patients who were still alive and free of relapse or NP at the 5-year post-operative mark (5-year event-free survivors, 5y-EFSs). This restricted cohort was used to estimate 10-year OS, IR, and INP conditional on having survived event-free until 5 years.

Univariate and multivariate Fine–Gray subdistribution hazard models for competing risks were performed to estimate subdistribution hazard ratios (SDHRs) with 95% confidence intervals (95% CIs) for 10-year IR and INP within the subset of patients with potentially 10 years of surveillance, by considering death from another cause (non-relapse or non-NP mortality, respectively) as the competing event. The models were adjusted for relevant clinical and pathological variables to identify independent prognostic factors for late events. Statistical analyses were conducted by a qualified expert statistician (F.S.) and performed using SAS Studio v. 9.04.01 statistical software.

## 3. Results

### 3.1. Patient Characteristics

Detailed patient characteristics are summarized in Table 1. The median age at surgery was 68 years, and the majority were males (612, 80.6%). Pathological stage IA represented 71% (539 patients) of the cohort, with adenocarcinoma being the most common subtype (500, 65.9%). The most common surgical procedure performed was lobectomy (599 patients, 78.9%). Adherence to the systematic follow-up protocol was high (98%). LNs, either pre- or post-surgery, were a frequent finding, affecting 60% (N = 456) of the total cohort, with 35.4% (N = 269) developing LNs post-surgery.

### 3.2. Overall Survival

**Figure 1:**
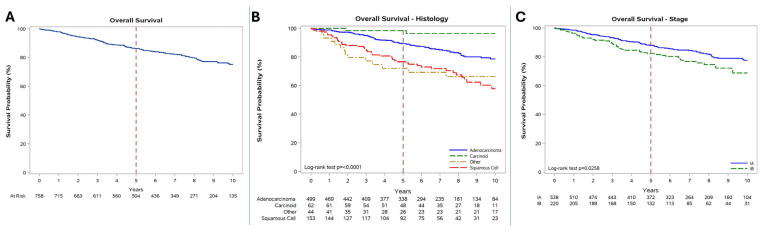
(**A**) Overall survival (OS) in the total cohort; (**B**) OS according to histology; (**C**) OS according to stage.

Median follow-up time was 6.5 (3.8–9.2) years. The estimated 10-year OS rate for the entire cohort was 75% (5-year OS: 86%). Ten-year OS was significantly higher in patients with a carcinoid histology (96%) than in those with adenocarcinoma (79%) or squamous-cell carcinoma (58%, *p* < 0.0001). A better 10-year OS was observed in stage IA patients (78%) compared with stage IB patients (69%; *p* = 0.0258, Figure 1).

### 3.3. Incidence of Relapse

The estimated 10-year IR for the entire cohort was 18% (1.8%/year; 5-year IR: 14%). Ten-year IR was significantly lower in patients with carcinoid (9%) than in those with adenocarcinoma (18%) or squamous-cell carcinoma (22%, *p* = 0.0257). A similar 10-year IR was reported in both stage IA (17%) and stage IB patients (22%; *p* = 0.3101).

Multivariate competing risk analysis for IR (Table 2) identified age (SDHR: 1.03, 95% CI: 1.00–1.06, *p* = 0.0357) and pack-years (SDHR: 1.01, 95% CI: 1.00–1.02, *p* = 0.0203) as the most significant risk factors.

**Figure 2:**
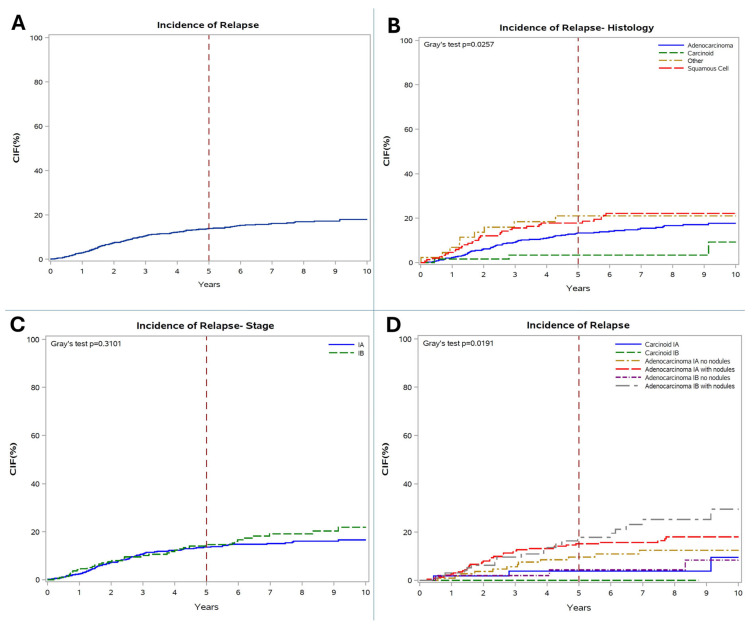
(**A**) incidence of relapse (CIR) in the total cohort; (**B**) IR according to histology; (**C**) IR according to stage; (**D**) IR according to histology and presence of lung nodules.

Subgroup analyses evidenced a significantly lower risk of relapse in patients with carcinoid IA/IB (0–10% at 10 years) and those with adenocarcinoma IA/IB without pre- or post-surgery LNs (8–12% at 10 years). These low-risk groups showed a similar low incidence of relapse (*p* = 0.5088), which was significantly lower when compared to patients with adenocarcinoma with LNs (p = 0.0191, Figure 2).

### 3.4. Incidence of New Primary Lung Cancer

The overall 10-year INP was 11% (average annual incidence rate of 1.1%). Patients with pre-surgery LNs (21% vs. 7%, *p* < 0.0001) and post-surgery LNs (17% vs. 7%, *p* < 0.0001) showed a markedly increased 10-year NP rate compared to those without nodules.

Multivariate competing risk analysis for INP (Table 3) identified LN presence (pre- or post-surgery) as the main significant risk factor (SDHR: 5.80, 95% CI: 2.23–15.11, *p* = 0.0003).

**Figure 3:**
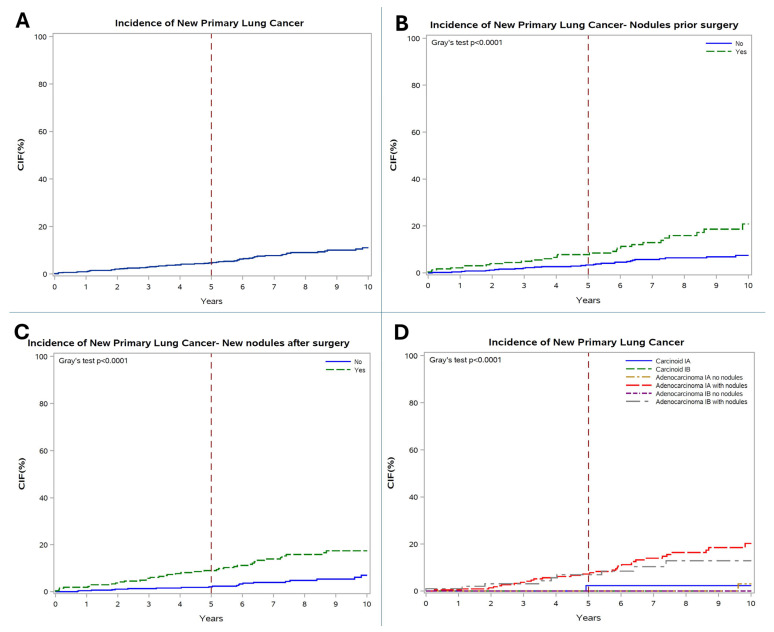
(**A**) Incidence of new primary lung cancer (INP) in the total cohort; (**B**) INP according to pre-surgery lung nodules; (**C**) INP according to post-surgery lung nodules; (**D**) INP according to histology and presence of lung nodules.

Subgroup analyses for NP demonstrated that a carcinoid histology (0-0.2%/year) and adenocarcinoma without LNs (0–0.3%/year) shared a statistically similar lower annual INP (*p* = 0.8062), which was significantly lower than the incidence observed in patients with LNs (*p* < 0.0001, Figure 3).

### 3.5. Five-Year Event-Free Survival Analyses

The landmark analysis was restricted to those patients (449, 59.1%) who were alive and free of relapse or NP at 5 years post-surgery (5y-EFS cohort). This subgroup experienced very favorable outcomes, specifically 10-year OS, IR, and INP of 92%, 5%, and 0.8%/year, respectively.

**Figure 4:**
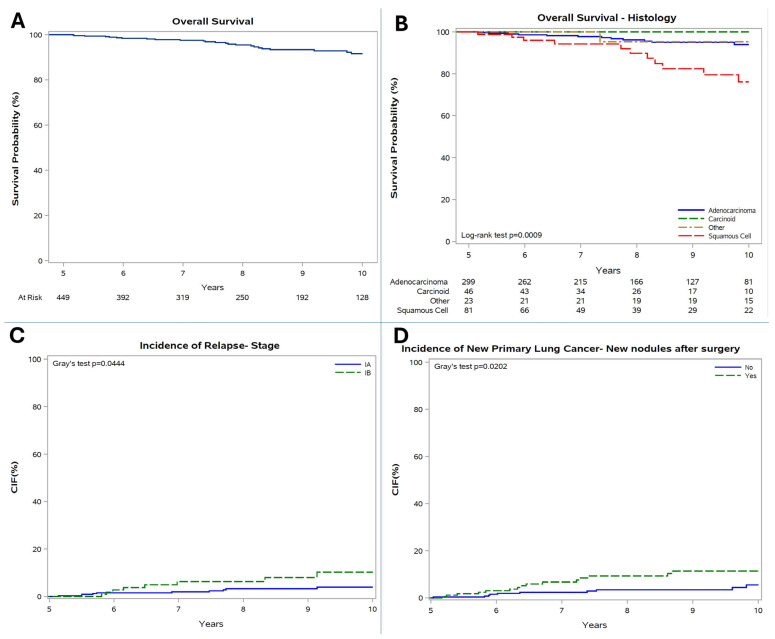
(**A**) Conditional OS; (**B**) conditional OS according to histology; (**C**) conditional IR according to stage; (**D**) conditional INP according to post-surgery lung nodules.

Subgroup analyses revealed that 10-year OS remained remarkably high for carcinoid (100%) and adenocarcinoma (94%) histologies, with a significant difference compared with squamous-cell carcinoma (76%) (*p* = 0.0009). Conditional 10-year IR was lower in stage IA (4%) vs. IB (10%, *p* = 0.0444). In addition, conditional 10-year INP was lower in patients with no pre-surgery LNs (0.5% vs. 1.5%/year, *p* = 0.0147) and no post-surgery LNs (0.6% vs. 1.1%/year, *p* = 0.0202, Figure 4).

### 3.6. Tailored Long-Term Surveillance Proposal

Based on the long-term event risk profiles, we propose a tailored surveillance strategy according to histology, stage, presence of LNs on CT scan, and 5y-EFS, grouping patients into three risk categories (low-, intermediate-, and high-risk; Table 4). This proposal was developed by exclusively integrating the variables (such as histology, pT stage, and the presence of LN) that were identified as independent and statistically significant prognostic predictors in the multivariate analysis for both the IR and INP. This approach is based on the finding that patients with lower-risk profiles do not experience a late increase in relapse or NP incidence, suggesting that less (or no) intensive surveillance can be safely adopted after the 5-year benchmark.

## 4. Discussion

The optimal surveillance strategy after curative surgery in early-stage NSCLC continues to be extensively discussed in different clinical societies [1,2,3,4], given the lifelong risk of NP and the economic burden of intensified follow-up [10]. Our study directly addresses this critical gap by analyzing a large single-institution cohort of completely resected pathological stage I NSCLC patients with extensive and standardized long-term follow-up, extending up to 10 years. We also used landmark analysis at the 5-year post-operative mark to precisely quantify the long-term risk conditional on surviving event-free for 5 years. Given this, the traditional 5-year benchmark for “cure” is routinely challenged by the persistent, lifelong risk of recurrence [5,11,12]. Moreover, multiple studies also support the concept of late recurrence—though less common than NP—further justifying the need for sustained vigilance [8,13,14,15]. In this setting, our findings confirm the utility of the 5-year time point for risk stratification. By isolating the 5y-EFS cohort, we demonstrated that conditional long-term outcomes (OS, IR, and INP) were significantly more favorable, confirming the concept of a ”delayed cure” for this specific subgroup. Crucially, the risk factors driving long-term events in this survivor group are often different from those predicting early recurrence [7,8]. In fact, while stage IB disease is associated with a higher absolute 10-year IR than stage IA (10% vs. 4%), suggesting that stage remains a relevant late-prognostic factor, the incidence of NP emerged as the dominating concern in the long term, with a conditional 10-year rate of 8% in the 5y-EFS group. As reported by other authors [8], the landmark analysis was essential to precisely quantify this conditional risk, providing a robust statistical foundation for a tailored approach.

Our results highlight that the presence of LNs—either pre- or post-operatively—is the single most powerful predictor for a late-stage event, specifically the development of NP. Patients with LNs showed a significantly higher 10-year INP (1.7–2.1%/year) compared to those without LNs (0.7%/year), and this high-risk profile persisted in the 5y-EFS cohort (1.1-1.5%/year vs. 0.5-0.6%/year). This finding strongly supports the concept of “field cancerization,” where the entire respiratory epithelium of high-risk individuals (often heavy smokers) is genetically or epigenetically altered, predisposing them to multiple primary tumors [16,17,18,19]. The presence of new or persistent LNs on CT scans may serve as a clinical decision-maker of this underlying high-risk state that reflects specific molecular events even in seemingly negative surgical margins [20]. These patients, often presenting with specific risk factors like high pack-years [5], require long-term, intensive monitoring due to this intrinsic biological risk.

Furthermore, we observed that squamous-cell carcinoma histology was associated with the lowest conditional 10-year OS (76%) compared to adenocarcinoma (94%), suggesting that histology remains a significant factor influencing late survival [7,11]. Conversely, carcinoid histology has the most favorable long-term prognosis (100% conditional OS), confirming that neuroendocrine tumors represent a distinct and low-risk group for late-stage events. Future studies are warranted to incorporate histology into the TNM staging system, given these results confirming the poorer survival for early-stage squamous-cell LC.

Despite the need for continued vigilance due to the risk of NP [1,3], current guidelines typically offer broad surveillance recommendations, often including indefinite annual CT scans. This rigid, “one-size-fits-all” model is consequently leading to clinical and economic inefficiency [10,21]. Moreover, a lifelong annual regimen may represent an ”over-surveillance”, leading to significant negative consequences like increased healthcare expenditures, “scanxiety”, and unnecessary patient exposure to invasive procedures driven by false-positive CT scan results. To overcome these issues, we propose a simple stratification of stage I patients into three distinct risk groups based on three easily accessible clinical parameters (histology, stage, and LN presence), promoting enhanced adherence to surveillance [22,23] by tailoring the burden. Low-risk patients (carcinoid; adenocarcinoma IA without LNs) show an extremely low conditional NP rate (0 to 0.2–0.3%/year), suggesting that cessation of annual CT scans at 5 years is a safe and feasible option. Continuing highly intensive surveillance in this group yields minimal clinical benefit while imposing unnecessary cost and patient anxiety [22]. Similarly, intermediate-risk patients (adenocarcinoma IB without LNs or 5y-EFS squamous-cell carcinoma stage IA without LNs) show a moderate but manageable conditional IR (0.8%/year) and NP risk (0.6%/year). In line with suggestions from other researchers [23], our proposal advocates for biennial CT scans extending to 10 years. This specific approach represents a tailored strategy that could help (1) to balance the need for early detection against the advantages of reduced follow-up intensity and (2) to reduce concerns regarding long-term adherence [24]. Conversely, high-risk patients (adenocarcinoma IA or IB with LNs and squamous-cell carcinoma) maintain both a high NP incidence (1.1–2%/year) and IR (up to 2.9%/year). As advocated by other authors [14,15,25], these specific patients must continue annual CT scans for up to 10 years to ensure the timely detection of new lesions (relapse or NP) amenable to curative treatment. This risk stratification model aligns with emerging evidence advocating for personalized follow-up in thoracic oncology [23] and provides clear criteria lacking in existing guidelines. Furthermore, real-world clinical practice studies often demonstrate low adherence (reported to be as low as 61.4% for the Medicare population) [24] to post-treatment surveillance guidelines. This non-compliance is frequently rooted in the clinical belief that follow-up intensity does not significantly improve patient outcomes. This creates a vicious cycle that our proposal aims to break: by offering a data- and risk-stratified de-escalation strategy, surveillance is made more targeted and rational, thereby potentially increasing physician compliance with a revised, evidence-based schedule.

Our study has several limitations inherent to its retrospective, single-institution nature. The cohort, while large, may be subject to selection biases. We acknowledge the lack of an a priori power calculation, which is a common feature of studies utilizing real-world data. Nevertheless, we emphasize that our single-center cohort size is substantial and, crucially, the long follow-up period (up to 10 years) enhances the power to reliably capture late events, which are the focus of our analysis. While analysis by resection type was performed, the findings were not statistically significant for OS or IR, leading to its omission from the primary focus of the manuscript. Since we did not report patients’ molecular profiling, we were not able to assess the impact of specific mutations (e.g., EGFR or ALK rearrangement), which may influence ultra-late recurrence in some cases [26]. Future research should focus on validating this simple clinical risk model in external, prospective cohorts. Furthermore, the integration of molecular markers (e.g., mutational signatures in NP, biomarkers from adjacent lung tissue, ctDNA) [19,20] with clinical data will be essential to further refine the low-risk subgroup, moving towards truly individualized surveillance protocols.

## 5. Conclusions

The risk of late-stage events in pathological stage I NSCLC survivors is heterogeneous and driven primarily by the risk of developing NP. By utilizing landmark analysis, we have defined distinct subgroups among 5y-EFSs, where the conditional risk of NP varies significantly based on histology and the presence of LNs. Our proposal for a tailored surveillance strategy offers a simple and clinically actionable risk stratification tool that can be easily implemented in dedicated follow-up clinics, safely de-escalating surveillance for low-risk patients while maintaining intensive monitoring for high-risk individuals. Future work integrating genomics and minimal residual disease with this clinical model will further optimize long-term follow-up and resource utilization.

## Figures and Tables

**Table 1:** Clinicopathological characteristics of the entire cohort.

		Patients (N = 759)
**Age**	Median (IQR)	68 (61–74)
**Gender**	Males	612 (80.6%)
	Females	147 (19.4%)
**Smoking (pack-years)**	Median (IQR)	33.3 (3.5–50)
**Prior cancer (except LC)**	N (%)	278 (36.6%)
**COPD**	N (%)	431 (56.8%)
**Familiarity**	N (%)	232 (30.6%)
**Lung nodules (LNs)**	N (%)	456 (60%)
	Pre-surgery LN	238 (31.4%)
	Post-surgery LN	269 (35.4%)
**Type of resection**	Lobectomy	599 (78.9%)
	Segmentectomy	122 (16.1%)
	Wedge	24 (3.2%)
	Bilobectomy	11 (1.4%)
	Pneumonectomy	2 (0.3%)
	Other	1 (0.1%)
**Stage (9th TNM ed.)**	IA	539 (71%)
	IB	220 (29.0%)
**Histology**	Adenocarcinoma	500 (65.9%)
	Squamous-cell carcinoma	153 (20.1%)
	Typical carcinoid	39 (5.1%)
	Atypical carcinoid	23 (3.1%)
	Carcinoma NOS	27 (3.5%)
	Large cell neuroendocrine carcinoma	8 (1.1%)
	Large cell carcinoma	6 (0.8%)
	Undifferentiated carcinoma	3 (0.4%)
**New primary LC**	N (%)	65 (8.6%)
Systematic follow-up *	N (%)	682 (98.0%)
**N. non-contrast CT scans**	median (IQR)	8 (6–10)
**N. contrast CT scans**	median (IQR)	4 (0–4.25)

* Biannual non-contrast chest CT scan and abdominal ultrasonography in the first 2 years, then annually till 10 years. LC: lung cancer; COPD: Chronic Obstructive Pulmonary Disease.

**Table 2:** Univariate and multivariate Fine–Gray subdistribution hazard models for competing risk for 10-year incidence of relapse.

		Univariate Model			Multivariate Model		
		Coefficient	SDHR (95% CI)	*p*-Value	Coefficient	SDHR (95% CI)	*p*-Value
Age	Continuous	0.03759	1.04 (1.01–1.07)	**0.0062**	0.03115	1.03 (1.00–1.06)	**0.0357**
Sex	Female vs. male	−0.18698	0.83 (0.45–1.55)	0.5556	0.02637	1.03 (0.55–1.90)	0.9333
COPD	Yes vs. no	0.01816	1.02 (0.63–1.64)	0.9408	−0.29168	0.75 (0.44–1.28)	0.2899
Pack-years	Continuous	0.00988	1.01 (1.00–1.02)	**0.0027**	0.00864	1.01 (1.00–1.02)	**0.0203**
Histology	Squamous-cell vs. non-squamous	0.67666	1.97 (1.21–3.21)	**0.0068**	0.49070	1.63 (0.97–2.74)	0.0629
Stage	IB vs. IA	0.219330	1.25 (0.75–2.07)	0.7154	0.18259	1.20 (0.71–2.02)	0.4917
Prior or new nodules	Yes vs. No	0.15383	1.17 (0.72–1.89)	0.5304	0.33993	1.41 (0.85–2.32)	0.1858

SDHR, subdistribution hazard ratio. CI, confidence interval. Event of interest: incidence of relapse. Competing event: death from another cause (non-relapse mortality).

**Table 3:** Univariate and multivariate Fine–Gray subdistribution hazard models for competing risk for 10-year incidence of new primary lung cancer.

		Univariate Model			Multivariate Model		
		Coefficient	SDHR (95% CI)	*p*-Value	Coefficient	SDHR (95% CI)	*p*-Value
Age	Continuous	0.02021	1.02 (0.99–1.05)	0.1129	0.02585	1.02 (0.99–1.06)	0.1491
Sex	Female vs. male	−0.20596	0.81 (0.37–1.78)	0.6056	0.02694	1.03 (0.46–2.32)	0.9482
COPD	Yes vs. no	0.58966	1.80 (0.93–3.49)	0.0794	0.09594	1.10 (0.54–2.24)	0.7906
Pack-years	Continuous	0.01240	1.01 (1.00–1.02)	**0.0092**	0.00992	1.01 (0.99–1.02)	0.0926
Histology	Squamous-cell vs. non-squamous	0.24997	1.28 (0.63-2.63)	0.4945	0.27184	1.31 (0.59–2.93)	0.5063
Stage	IB vs. IA	−0.13385	0.88 (0.42–1.83)	0.7225	−0.19824	0.82 (0.38-1.77)	0.6138
Prior or new lung nodules	Yes vs. No	1.71298	5.55 (2.16–14.22)	**0.0004**	1.75749	5.80 (2.23–15.11)	**0.0003**

SDHR, subdistribution hazard ratio. CI, confidence interval. NP, new primary lung cancer. Event of interest: incidence of NP. Competing event: death from another cause (non-NP mortality).

**Table 4:** Proposal for tailored surveillance according to risk group.

Group	10 Y—IR	10-Y IR (5yEFS)	10 Y NP	10 Y NP (5 yEFS)	Recommendation
CARCINOID IA	10%	6%	0.2%/year		Stop annual scans at 5 years
CARCINOID IB	10%	0%	0%/year		Stop annual scans at 5 years
ADC IA (NO LN)	12%	3%	0.3%/year	0.4%/year	Stop annual scans at 5 years
ADC IB (NO LN)	8%	5%	0%/year	0%/year	Consider biennial CT scan after 5 years
ADC IA + LN	18%	4%	2%/year	1.6%/year	Annual scans till 10 years
ADC IB + LN	29%	16%	1.3%/year	0.8%/year	Annual scans till 10 years
SCC	22%		1.1%/year	0.6%/year	Annual scans till 10 years
**HISTOLOGY**	**IA**	**IB**	**LN**	**5-y EFS**	**RISK GROUP**
CARCINOID	x		(x)		Low
		x	(x)		Low
			(x)	x	Low
ADC	x			(x)	Low
		x		(x)	Intermediate
	x		x	(x)	High
		x	x	(x)	High
SCC	-	-	-	-	High
				x	Intermediate (if stage IA and no LN)

ADC: adenocarcinoma; SCC: squamous-cell carcinoma; LN: lung nodules.

## Data Availability

Data is maintained within this article.
